# Information and communication technologies and gender in climate change and green economy: Situating women’s opportunities and challenges in Zambian policies and strategies

**DOI:** 10.4102/jamba.v8i3.243

**Published:** 2016-04-12

**Authors:** Justina Namukombo

**Affiliations:** 1 Department of Political and Administrative Studies, University of Zambia, Zambia; 2 Department of Literature and Languages, Geography and Environmental Education unit, Lusaka regional centre of expertise on education for sustainable development, Zambia

## Abstract

Zambia’s 2012 report on the United Nations Conference on Sustainable Development (RIO +20) identifies existing opportunities on the country’s transitioning to green economy. The RIO +20 conference of 2012 has resulted in new momentum in addressing problems of sustainable development. However, this article argues that there are practical challenges that require paying attention to, especially those involving women. The article addressed one key question: To what extent can women participate in the transitioning process to green economy in Zambia and what opportunities and challenges exists? The study used document analysis to answer the above question. National policy documents were reviewed to understand interventions on environmental management. Whilst going through the documents, the study used gender analysis frameworks (education, skills, roles in family and society, access to infrastructure) to bring out qualitative and quantitative information on women. Using suggested green economy interventions in the literature as benchmark, qualitative analysis was used to project possible participation of women in green economy activities and possible challenges to be faced. The study found that participation of women will be limited despite existing opportunities because of challenges of access to information and communication technology infrastructures, low educational levels and skills and financial constraints. As Zambia undergoes a transitioning process, these limitations should be addressed in planned green economy policies and interventions to maximise benefits.

## Introduction

A decade after the World Summit on Sustainable Development in Johannesburg in 2002, the world leaders again gathered at Rio de Janeiro in Brazil to look at the future of the environment. This time one of the themes was green economy, sustainable development and poverty eradication. Green economy was defined by the United Nations Environmental Programme as development that leads to social well-being and social equity whilst at the same time addressing effects on the environment (UNEP 2011). On practical part, green economy is development confined to low carbon, renewable and efficient energy technologies and environmentally friendly farming and fishing practices (Fulai 2009). Currently, governments are looking at the option of green economy as a way to prevent and mitigate effects of climate change and take on different approaches to development (Africa Progress Report 2014; UNECA 2012; UNESCO 2013).

Since then, most countries in Africa, Zambia inclusive, have been trying to contextualise the concept in local environmental interventions. In Zambia, attempts have been made through articulation in the RIO +20 Report by convening meetings by necessary ministries like Finance and National Planning, Ministry of Environment Tourism and Natural Resources and other stakeholders working in the area of environmental protection. The article forms part of the broader efforts to understand how green economy initiatives can be interpreted and contextualised in national policies and strategies and their implication. The article specifically focuses on implications of gender and Information and Communication Technologies (ICTs) in climate change and green economy using Zambia as a case study. The Zambian Report on the United Nations Conference on Sustainable Development (RIO +20) has already identified existing opportunities on the country’s transitioning to green economy. For example, the existence of the Renewable Energy Strategy whose objective is to promote measures aimed at investing in renewable sources of energy such as solar, biomass, wind and biofuels. National Climate Change Response Strategy (2011:ii) has a vision of ensuring that the most vulnerable sectors of the economy are climate proofed. For example, development of sustainable land use systems to enhance agriculture production and ensure food security. The National Climate Change Policy (2012:10–12) has put up adaptation and disaster risk reduction–related measures in most vulnerable sectors like water, agriculture, forestry, energy and infrastructure. In the water sector for instance, the aim is to enhance investment in water capture and storage such as dams, strategic boreholes and tanks, construct water basin transfers and improve drainage.

The article explores the position of women in the green economy transitioning process of Zambia. The key question raised is: To what extent can women participate in green economy initiatives? The analysis is performed using gender approaches to understand access problems for women including those of ICTs. Women have been sidelined in most development activities because of their low educational levels, poverty, sociocultural factors and access problems to physical infrastructure (Hafkin 2002). On the other hand, ICTs have been identified as having potential to facilitate the transitioning process to green economy (Ciocoiu 2011; Young 2011). ICTs include a ‘variety of analogue and digital technologies: telephones, radios, television and computers’ (Lotter 2007:3). Devices like radio, television and cellular phones can facilitate access to information and enable participation in development activities.

In Zambia, ICTs are intended to be part of climate-resilient technologies in various regions of the country (Zambia Climate Policy 2012). The Zambian national gender policy also intends to facilitate participation of women in development by promoting changes in patterns of socialisation and gender division of labour (Zambia Gender Policy 2004). There is no doubt that women would face similar challenges they have faced in prior development interventions. It is important to spell out these challenges and existing opportunities that are specific to green economy initiatives. This is important as countries contextualise the concept in their environmental management policies and strategies.

## Research methodology

The study addressed one key question: To what extent can women participate in the green economy transitioning in Zambia and what opportunities and challenges exist for them in this process? The study used document analysis to answer the above questions. A number of national policy documents were reviewed. Namely, nine national policy documents on environment were reviewed to understand interventions on environmental management and how women participation has been addressed; two policies on gender and ICT to learn on interventions aimed at addressing women needs and local reports on proposed ways of achieving green economy were also reviewed. The process involved first purposively looking for local policies and regulations on environment, review documents (local and international) including journal articles making suggestions on how to achieve green economy. The study also made an extensive review of statistics on access and use of ICTs by women, access to education with specific focus on ICT subjects and other barriers which women face. Information on suggested ways of achieving green economy is later used to project possible participation of women in green economy activities.

Document analysis has been used before in environmental and climate change studies. For example, Nhamo (2014:3) used document analysis in his journal article ‘addressing women in climate change policies: A focus on selected east and southern African countries’. He critically analyses climate change policy documents from selected countries to establish his empirical evidence on his topic. Document analysis is therefore a credible method of data collection. However, it has challenges of biasness as the researcher will most likely not include documents with opposing views to their study. Table 1 shows the policy documents used as sources of information. With these limitations in mind, the study included international documents to supplement local sources of information.

**TABLE 1:** Reviewed policy documents.

Number	Policy document reviewed	Type of information collected
1.	National Climate Change Policy (2012)	Interventions on climate change
2.	National Climate Change Response Strategy (2011)	Interventions on climate change
3.	National Energy Policy (2007)	Sustainable use of energy
4.	*Mines and Minerals Amendment Act* of (2007)	Management of wastes from mineral activities
5.	National Policy on Environment (2006)	Intervention on environmental management
6.	National Information and Technology Policy (2006)	Provision and access to ICT infrastructure
7.	Living Conditions Monitoring Survey (2006 and 2010)	Socio-economic conditions between men and women
8.	National Solid Waste Management Strategy (2004)	Interventions on solid waste management
9.	National Gender Policy (2004)	Interventions to address gender inequalities
10.	*Environmental Protection and Pollution Acts* (1990 and 1997)	Guidelines on release and management of pollutants
11.	FAWEZA Report (2011)	Zambian situation on access to ICT subjects between girls and boys
12.	United Nations Economic Commission for Africa (UNECA 2010)	Suggestions on how to achieve green economy
13.	Zambian Workshop Report on Inclusive Green Growth in Zambia (2014)	Suggestions on how to achieve green economy
14.	UNESCO (2013)	World statistics on access to education and literacy levels

## Findings and discussion

### Zambian context: Climate change and green economy

Zambia has a population of 13 million people, and it is growing at a rate of 2.9% per annum. Women in Zambia make up 51% of the population. According to the Zambian Living Conditions Monitoring Survey (LCMS), about 64% of the population live in poverty (LCMS 2010). Only 22% of the country’s population has access to electricity (Zambia Energy Policy 2007). Zambia is also endowed with vast natural resources and 60% of its land is covered by forest. However, the country faces environmental problems. For instance deforestation has been responsible for loss of about 250 000 ha – 300 000 ha of forest per annum (Zambian National Policy on Environment 2006). In 2000, Zambia is reported to have produced about 54 718 metric tons of carbon dioxide, and estimates are that between 2000 and 2030, emissions are expected to increase from 54.718 metric tons to 216.8 million (Zambia RIO +20 UN report 2012). This has led to manifestation of some effects of climate change. Based on country assessments and also international assessments, effects of climate change have been brought to light. The Zambia Metrological Department report increases in frequency of extreme events like floods and droughts over the four decades and emerging tendency of delayed onset and earlier ending of rainfall (Zambia National Climate Change Response Strategy 2011). UNDP report on Zambia’s climate change profile indicates increase in annual temperatures by 1.3 °C since 1960 and increase is at an average rate of 0.29 °C per decade (Zambia National Climate Change Response Strategy 2011). Table 2 shows the occurrence of natural disasters for the indicated periods.

**TABLE 2:** Occurrence of natural disasters from 1980 to 2009.

Natural disasters	1980–1989	1990–1999	2000–2009
Droughts	2	2	1
Floods	1	2	11
Storms	0	0	0
Emergency ‘events’	3	4	12

*Source:* Republic of Zambia, Ministry of Tourism, Environment and Natural Resources, National Climate Change Response Strategy (NCCRS:13)

From the above described effects of climate change, there is no doubt that Zambia needs to work at any mechanisms that are believed to avert this situation.

The United Nations RIO +20 Zambia report identified pathways for Zambia’s implementation of green economy. The basis upon which Zambia is adopting the green economy agenda is the many environmental management policies and regulations that the country has been implementing. To date, Zambia has about 33 legislations and a signatory to about 21 international conventions on environmental protection (Environmental Council of Zambia 1994). In addition, the Sixth National Development Plan and Vision 2030 have articulated how development will be made sustainable through implementation of many sectoral policies and programmes. In other words, Zambia has some aspects of the ‘first’ green economy readiness as Nhamo (2013) described it. However, there is a need to postulate how women will be able to participate in green economy initiatives in the context of the many challenges they have faced before including their recent access to and use of ICTs.

In Zambia, the first initiative to explore on green economy began with the African Development Bank workshop cosponsored by the Organisation for Economic Development in January, 2013. The workshop was on ‘Green Growth in Africa’ and was held in Lusaka, the capital city. As a follow-up to the earlier workshop, Ministry of Finance and National Planning and Ministry of Lands, Natural Resource Management and Environmental Protection^1^ jointly organised a workshop on ‘Inclusive Green Growth’ (IGG) from 04 to 05 July 2013. The workshop was attended by 26 participants from government, private sector, academic and research institutions and civil society.

From the workshop, participants attempted to come up with a definition of what green economy or IGG meant in the Zambian context. IGG was defined as ‘inclusive development that makes sustainable and equitable use of Zambia’s natural resources within ecological limits’ (Banda & Bass 2014:3). The workshop participants made suggestions on how in the Zambian context development activities could be tailored in the green economy direction. The following suggestions were made:

- investing in natural resources that can make money for the poor
- investing in people’s capacity to combine green and inclusive approaches
- long-term perspectives to build institutional and economic resilience as well as financing models that are more ‘patient’ for their returns
- making business houses and civil society take the lead
- focus on both projects and governance. That is working on the institutional framework: policy, finance and enabling environment (Banda & Bass 2014:11).

The ultimate output of the workshop was to produce an operational Zambian IGG Strategy. Because this article relied on secondary sources of information, it was not possible to establish if the strategy has been formulated. The suggestion is to carefully take into account women’s contribution and the challenges they would face.

Apart from these recent articulations Zambia has been implementing a number of policies and has enacted a number of regulations with regard to environmental protection. These could be used to start specific programmes and projects aimed at achieving objectives of the green economy. Table 3 highlights some of the policies and regulations and their possible contribution to achieving IGG or green economy.

**TABLE 3:** Policies and regulations on environmental protection in Zambia and their possible contribution to achievement of Inclusive Green Growth or green economy.

Number	Policy or regulation	Objective or focus area	Possibility of contributing to IGG
1	2006 National Policy on Environment	Provide framework for management of Zambia’s environment and natural resource in order to retain their integrity to support need of current and future generation	Sustainable management of resources would help achieve aspirations of IGG
2	National Climate Change Policy (2012)	Climate change–specific interventions in crucial sectors, e.g. energy, agriculture, mining, forestry etc	Promotion of utilisation of renewable energies, use of cleaner technologies, promotion of sustainable use of land
3	National Energy Policy (2007)	Ensure availability of adequate and efficient supply of energy from sources that are dependable, low cost and environmentally friendly	IGG is looking for possibilities of using clean and efficient sources of energy, which will reduce emissions of carbon and other Green House Gasses
4	*Mines and Minerals (Amendment) Act* of 2007	All applications for mineral processing licenses to be accompanied by an environmental management plan	With adequate enforcement, potential effects on the environment by mining processing can be reduced
		Plan includes proposal for the prevention of pollution, treatment of waste, protection and reclamation of land water resources	
5	*Environmental Protection and Pollution Acts* of 1990 and 1997	Ensuring developers submit project briefs and environmental impact assessment specifying products to be produced: solid, liquid, gaseous, waste generation and other emissions	Control of harmful emissions into the environment contribute to reduction of harmful wastes liquid or gaseous accumulating
6	National Solid Waste Management Strategy for 2004	Provide a strengthened framework on management of waste	One of the ways to reduce carbon emissions and other greenhouse gases is through recycling of waste

### Theoretical positioning of women in transitioning process

Having looked at the policies and regulations on environmental management and also suggestions being made on how to achieve green economy, it is possible to position women in this transitioning process. This is presented as a framework in Table 4 by showing the extent to which women can participate and the possible challenges that can be faced.

**TABLE 4:** Theoretical positioning of women’s participation in green economy initiatives and expected challenges.

Requirement for transitioning	Opportunities for women participation	Possible challenges to be faced
Energy serving systems: Transforming ways through which energy is generated and consumed. Transforming ways in which we use water and grow food.	Women use energy and other resources for their cooking requirements and other household chores.	Women have always faced a challenge of being left out in making of decisions, do not own land and therefore may not be able to make decisions on how land is used.
	Most agricultural work especially manual labour is carried out by women.	Will need information on why they need to adopt new practices of using energy, water and other natural resources
Sustainable use of natural resources: Agricultural practices that reduce water pollution, soil erosion, increase use of organic inputs	Mostly women are used as labourers in agriculture, therefore are better positioned to implement initiatives aimed at using natural resources sustainably.	Women will need information on why they need to adopt new practices of using energy, water and other natural resources.
		Will only implement what has been decided by land owners even at family level.
Decarbonising the economies Recycling waste Generating electricity from land-fill gas Restoring the soil Managing grasslands and soils in a sustainable way	Because of high poverty levels amongst women, they are the ones who mostly depend on unsustainable sources of energy like charcoal. They make good target for initiatives on alternative sources of energy.	High poverty levels amongst women can prevent them from adhering to initiatives aimed at managing natural resources sustainably
	Women also earn a living from scavenging from dump sites. Recycling initiatives can involve them.	
Skills for green jobs	In the process of greening the economy, new technologies that enable efficient use of resources are very important.	Women have lagged behind both in enrolment of ICT subjects and in use of ICTs. This will prevent them from being involved as they will not have the necessary skills.
Policies and legislation	Policies and legislation stipulate direction in which a country intends to take on specific interventions. Policies should aim at addressing challenges that would prevent women and men from participating.	Participation of women in policy making has always been a challenge.
Programmes and projects	Specific projects could target women especially those aimed at giving information on sustainable use of natural resources.	Most women are illiterate and may not understand English the language through which most information is communicated.
		Women may not own radios, television or access Internet through which information is communicated.

### Gender implications of transitioning to green economy: Situating women’s opportunities and challenges

#### Women’s access to information and communication technologies and possible participation in green economy

Access to information in Zambia is through both the electronic and print media. Electronic media is through television, telephones, Internet/emails, whilst print media is through newspapers, magazines and posters. Television services are offered by government and private sector. Three broadcasting stations are operational: namely Zambia National Broadcasting Corporation (ZNBC), Trinity Broadcasting and Muvi TV. The government broadcasting services (ZNBC) are found in every province of the country. The general challenge in the electronic media is limited coverage across the country (Zambia ICT Policy 2006).

As of 2004, Zambia had three licensed mobile cellular providers: Zamtel, MTN and Air Tel. By the same year, Zambia also had 300 telecentres offering telephone and email or Internet. Access was in major urban centres with large coverage along the line of rail. Though there has been increase in access to ICTs from 2004 to 2010, very few people own ICT assets. Table 5 shows increases in ownership from 2004 to 2010 between rural and urban areas.

**TABLE 5:** Trend in access to Information and Communication Technologies assets from 2004 to 2010 in percentages.

Asset	2004			2006			2010		
		
	All	Rural	Urban	All	Rural	Urban	All	Rural	Urban
Television	27.1	6.9	50.8	24	7.8	54.6	29.7	12.3	60.9
Radio	54.4	43.2	67.7	55.6	50.1	65.8	47.4	42.6	56
Land telephone	3.2	0.4	6.4	1.2	0.2	3.2	0.7	0.1	1.6
Cellular phone	10.8	1.9	21.2	24.2	8.8	53.1	49.4	32.4	80
Internet connection	0.3	0.1	0.5	-	-	-	-	-	-
Satellite dish/decoder	1.6	0.3	3.1	3.6	0.7	0.9	10.8	3.2	24.5

*Source:* Republic of Zambia (2012), Zambian Living Conditions Monitoring Survey Report for 2006 and 2010. Can be accessed on http://www.zamstats.gov.zm

Ownership in television sets slightly increased from 27.1% in 2004 to 29.7% in 2010. Increases were also experienced between the rural and urban areas with urban areas having significant improvements. During the same period under review, 32.4 of the rural population had mobile phones compared to 80% in urban areas. According to statistics provided by the Zambia Information Communication and Technology Authority (ZICTA) on their website, as of third quarter of 2014, there were 9316 mobile cellular subscriptions and 3 362 056 mobile Internet users and 23 fixed Internet subscriptions (ZICTA 2014). However, there are differences in access of ICTs between men and women. Table 6 shows these discrepancies.

**TABLE 6:** Ownership of Information and Communication Technologies assets by sex.

Asset	2004			2006			2010		
		
	All	Rural	Urban	All	Rural	Urban	All	Rural	Urban
Television	27.1	29.1	20.3	24	25.8	18.4	29.7	31.7	23.1
Radio	54.4	14.8	8.8	55.6	61.6	35.5	47.4	52.5	30.5
Land telephone	3.2	3.4	2.3	1.2	1.3	1	6.7	0.7	0.5
Cellular phone	10.8	11.5	8.2	24.2	26	18.5	49.4	51.5	42.5
Internet connection	-	-	-	-	-	-	-	-	-
Satellite dish/decoder	1.6	0.6	0.5	3.6	4	2.3	10.8	11.7	7.9

*Source:* Republic of Zambia (2012), Zambian Living Conditions Monitoring Surveys for 2006 and 2010. Can be accessed on http://www.zamstats.gov.zm

As can be seen from the table, ownership amongst women of the most commonly used sources of information in Zambia (television, radio and cellular phones) is poor. For example, the 2010 survey shows 31.7% of men having television sets compared to 23.1% of women. By 2010, only 42.5% women owned mobile phones compared to 51.5% males. Such poor access of ICT amongst women would limit participation in interventions on green economy. The Research ICT Africa survey results (as cited in Gilwald, Milek & Stork 2010) for 2007 had similar findings. According to the survey, Internet is accessed and used differently between men and women. Apart from Cameroon, the rest of the countries including Zambia had more men than women claiming to know what Internet is and having email addresses. In the country-level analysis amongst the 17 countries where the survey was conducted, Zambia had about 71% of the men listening to the radio compared to 45% of the women. For television, men almost double (48% against 26%) the number of women who are able to watch television. About 58% of the men were also found to own a mobile phone or active Subscriber Identity Module card as compared to 37% of the women.

#### Women education and skills and possible participation in green economy

There has been differential access to education for men and women in Zambia. This has resulted in more women not being able to read and write as compared to men. About 77% of the men are able to read and write compared to 58% of the women (Republic of Zambia 2006). Data on access to education reported by the Zambia Living Monitoring Conditions Survey show that overall, gross attendance rates increased for primary grades 1–7, from 105% in 2006 to 108% in 2010. The secondary gross attendance rate (grades 8–12) increased from 55% in 2006 to 64% in 2010. However, in both years gross attendance rates for boys were consistently higher than those for girls (LMCS 2010:63). Though participation and access to science, mathematics and technical subjects has been improving between male and female pupils, some schools still have more males enrolled in these subjects. A survey conducted in Zambia by the Forum for African Women Educationalists in Zambia on access and participation of girls in science, mathematics and technical subjects highlight some of these discrepancies (FAWEZA Report 2011:33). Table 7 shows the numbers of girls and boys taking science, mathematics and technical subjects in surveyed technical schools.

**TABLE 7:** Girls and boys taking science, mathematics and technical subjects (combined).

School	David Kaunda	Hilcrest	Kambule	Ndola Technical	Chiwala	SOLTEC	Mungwi	Linda
Girls	6	57	40	24	2	24	24	36
Boys	17	60	25	n/a	13	12	12	19

*Source:* Forum for African Women Educationalists in Zambia (2011) Situational Analysis Survey Report

In the context of climate change and green economy, it has to do with ability to use various ICTs and access digital information. It is about understanding the use of ICTs and being able to operate them. For one to be able to use ICTs, computer skills, information literacy and language will be very important. Because most women are less likely to be literate, this could be a barrier to use of ICTs. According to UNDP (as cited in Huyer 2003), literacy is in various types: functional literacy which enables a person to perform daily life activities with less difficulties, for instance, the ability to read newspapers, books and pamphlets. Functional literacy will enable one to operate a cellular phone or Internet connection. Medium of communication in using ICTs is usually in English which most illiterate people are not able to understand. This effect extends to the ability to read newspapers, books and pamphlets. With so many women not being able to read and write, this means being left out of the whole system. Scientific literacy enables one to respond to everyday issues in an informed manner. These include decisions on sources of energy, preservation and use of natural resources and ability of communities and families to make appropriate decisions concerning resource allocation, diet and sanitation and community development.

#### Financial resources amongst women and their participation in green economy

In Zambia, more female-headed households are considered poorer than male-headed households (57% compared to 49%). Average monthly earnings between men and women also differ. In 2005, a Zambian woman earned on average ZBK 196 453 (approximately $ 242) compared to a man’s earning of ZBK 354 453 (approximately $ 506) (LCMS 2006). Usually, women have less access and control over resources. For instance, resources in agriculture regarded important for production like land, equipment and inputs are usually owned by men (World Bank 2004). Initiatives on green economy will require women having access to various information on green economy and access to energy-saving technologies.

### Opportunities

Despite the above discussed challenges, there are a number of opportunities for women to participate in climate change and green economy:

- Women in Zambia constitute over 51% of the population. Initiatives should therefore target them as they will be assured of reaching greater numbers.
- In the agriculture sector, studies in Zambia have shown that women spend more time carrying out agricultural activities than men. For example, women spend 53% of total hours in agriculture work compared to the 47% spent by men (World Bank 2004:19).
- The poor in general are considered to have high dependence on nature for their livelihoods. Women are usually in worse condition with regard to poverty levels. Initiatives on protecting the environment can therefore be more appealing to them.
- Women in Zambia are also amongst populations that earn their livelihood from scavenging from dump sites. Projects on recycling can therefore involve them.
- Women are most likely to be involved in alternative sources of energy as they are the ones who use charcoal as a source of energy.

## Conclusion

The article has reviewed suggested requirements for transitioning to green economy both at the international and regional level. Zambian early perspectives on the concept of green economy have also been captured. That is, investing in natural resources that can make money for the poor, investing in people’s capacity, long-term perspectives to build institutional and economic resilience, making business houses and civil society take the lead and focusing on both projects and governance. In addition, Zambian policies and strategies on environmental protection have also been reviewed with an aim of pointing out their possible contributions to green economy aspirations. Parallel to these discussions, factors limiting the involvement of women in development in general have been discussed. These have been used to postulate possible or no participation of women in the green economy interventions. The article argues that women participation in green economy interventions will be limited as they have problems of access to ICT infrastructures, low levels of education, lack skills and have constraints of financial resources. With these challenges, Zambian situation has been used to substantiate these arguments and the article argues that green economy interventions should have in mind these limitations, for example, low education levels amongst women compared to that of men, low enrolments in science, mathematics and technology subjects, low access to assets commonly used as channels of communication like mobile phones, radios and televisions. However, opportunities have also been identified for possible participation of women in the green economy initiatives: they spend more hours in agricultural activities and are users of unsustainable sources of energy like charcoal. This makes them better targets for implementation of green economy activities.
